# A Novel Mini-Channel Heat Sink Design with Arc-Type Design Domain by Topology Optimization

**DOI:** 10.3390/mi13020180

**Published:** 2022-01-25

**Authors:** Tao Zhou, Chao Guo, Xiaodong Shao

**Affiliations:** School of Electromechanical Engineering, Xidian University, Xi’an 710071, China; taozhou@stu.xidian.edu.cn (T.Z.); cguo_01@stu.xidian.edu.cn (C.G.)

**Keywords:** topology optimization, design domain, nusselt number, pressure drop

## Abstract

Based on the advantages of the topology optimization method, a new mini-channel heat sink with arc-type design domain topology design is proposed in this work. This arc-type design domain is used to realize the flow distribution uniformity. Two dual objective optimization functions were selected to complete the topology design, and two topology optimization mini-channel models M1 and M2 were obtained. The aim of M1 is to achieve minimum average temperature and fluid dissipation of the heat source area. The fluid dissipation was used to characterize the pressure drop characteristics. The aim of M2 is to achieve minimum temperature difference and fluid dissipation of the heat source area. Then, the fluid and heat transfer characteristics of M1, M2, and the traditional straight heat sink M3 were analyzed by numerical simulation. Compared with straight mini-channel heat sink M3, the temperature differences of the mini-channel heat sink designed M1 and the mini-channel heat sink designed M2 were decreased by 31.6% and 42.48%, respectively. Compared with M3, the pressure drops of M1 and M2 were decreased by 22.7% and 30.9%, respectively. Moreover, the Nusselt number of the mini-channel heat sink designed M1 was increased by 34.43%. In comparison, that of the mini-channel heat sink-designed M2 increased by 15.86%. The thermal performance evaluation criteria (PEC) showed that the PEC value of M1 was greater than 1.4, while the PEC value of M2 was less than 1.14. Finally, experiments were conducted for M1 to verify the accuracy of the numerical simulation. It was found that the simulation results agreed well with the experimental results.

## 1. Introduction

The development of microelectronics technology makes electronic equipment becoming smaller, more integrated and more intelligent. This leads to the electronic equipment suffering higher heat flux, which can seriously affect the life and performance of electronic chips. Therefore, the realization of efficient heat dissipation of electronic chips has become an important topic of today’s research [1,2,3,4]. As an efficient heat transfer device, mini-channel heat sinks have gotten wide application.

Theoretical method [5,6] and numerical simulation method [7,8,9,10,11,12] are the two most common ways to study the cooling ability of heat sinks. In the field of heat transfer, some new theoretical research methods are proposed. Moradikazerouni et al. [13] proposed a CFD node calculation method for the flow of cryogenic fluid in the tank, and studied the influence of the physical characteristics of the tank on Rayleigh Bernard convection. Ma et al. [14] studied the natural convection heat transfer of nanofluids (water/Al_2_O_3_) in an inclined square enclosed space by using the finite volume method (FVM). Their results showed that nano additives increase the heat transfer rate. Estebe et al. [15] proposed a low Mach number adaptive grid method to simulate the phase transition of multiphase flow in cryogenic fuel tank. Moradikazerouni et al. [16] proposed a novel 0D/3D method to simulate a closed cylindrical tank driven by natural convection. Their results showed that this method can explain the different hydrodynamics and heat transfer, and can deduce low dimensional performance models suitable for different applications. These latest research in this field have made great contributions to the research of heat transfer and hydrodynamics.

In recent years, based on the experience of researchers, the structural optimization of the traditional heat sink model has also been proposed. Vafai et al. [17] proposed a multilayer channel heat sink (MCHS). This multilayer mini-channel fins have a lower thermal resistance compared to single-layer mini-channels. Deng et al. [18] designed an entrant mini-channel and found that the average Nusselt number was increased by 39%, the total thermal resistance was decreased by 22% and the pressure drop loss was considerable compared to the traditional rectangular channel. Moradikazerouni et al. [19] studied the thermal performance of air-cooled flat plate heat sink under forced convection boundary conditions. Their results showed that increasing the number of fins and fin height of the heat sink reduced the surface temperature. These structural optimizations significantly improved the heat transfer capacity of the heat sink. However, this traditional design idea still limits the design of the model.

The topology optimization method is an effective method for the heat sink design, which obtains the channel structure corresponding to the optimal performance by changing the material layout in the design domain. This method breaks through the design defects of the traditional heat sink, adaptively arranges the heat sink structure, and greatly improves the heat exchange capacity. In 1988, Bendsøe and Kikuchi [20] firstly proposed topology optimization technology and obtained the optimal configuration. For the first time, Borrvall and Peterson [21] established an optimal model with the minimum power consumption as the optimal goal to get the best channels. Dede et al. [22] used COMSOL and MATLAB commercial software to study the topology optimization of fluid flow and heat transfer. Zhang et al. [23] obtained a 2D nanofluid mini-channel heat sink (NMHS) by density-based topology optimization method. Joo et al. [24] obtained the optimal design of mini-channel heat sink under natural convection conditions by topology optimization. Han et al. [25] used topology optimization method to complete the design of cobweb design domain. They showed that, compared with the traditional cobweb structure, the temperature difference of the topological channel was reduced by 57.32%. Qiu et al. [26] realized the topology optimization design and found that, compared with the straight channel, the velocity distribution uniformity of the optimized heat sink is increased by 61.1%. Zhou et al. [27] proposed a topology optimization design with the three inlets and one outlet design domain. Their results showed that the temperature difference of the optimized radiator is reduced by 62.5% compared to the straight channel heat sink. However, most of the performance of the mini-channels obtained topology optimization are studied by simulation and theoretical implementation, which are rarely verified by experiments.

The fluid manifold can achieve the consistency of flow velocity in parallel flow channels, which ensures the uniformity temperature of the heat sink bottom [28,29,30]. This leads to the development of the heat sink design. However, the application of fluid manifold in topology optimization is relatively few. In order to achieve better velocity uniformity in the design domain, Li et al. [31] used topology optimization method to study the flow distribution problem that generates the flow field with optimal uniformity by setting a specified boundary with flow equality constraint to distribute the flow. Hu et al. [32] used a topology optimization method to get the triangular-type design domain under non-uniform heat source.

In this paper, a new type heat sink is designed by topology optimization. The rectangular design domain with the arc-type domain is proposed. The arc-type design domain locating at the inlet and outlet permit that all the flow path are similar approximately, that is, the flow resistance at different positions from the inlet to the outlet in the whole design domain is equal. According to the topology optimization design theory, this new type heat sink with arc-type design domain can be obtained.

The main innovations of this work include: (i) A novel heat sink design is achieved by topology optimization method. (ii) Arc-type design domain realizes better uniform flow distribution.

## 2. Problem Description

In this paper, topology optimization method is used to produce a novel heat sink. Fluid-structure coupling heat transfer problem is solved by coupling the flow field and temperature field, and the Brinkman penalty model [21] is used to realize the redistribution of fluid and solid in the design domain. The arc-type design domain is used to realize the flow uniformity in the topology process. The 2D topology structure is extended to the 3D topology mini-channel heat sink. The flow and heat transfer characteristics of the 3D topological mini channel heat sink is analyzed by numerical simulation. Finally, experiments are completed to verify the accuracy of the simulation.

## 3. Model and Topology Optimization Design

**Figure 1:**
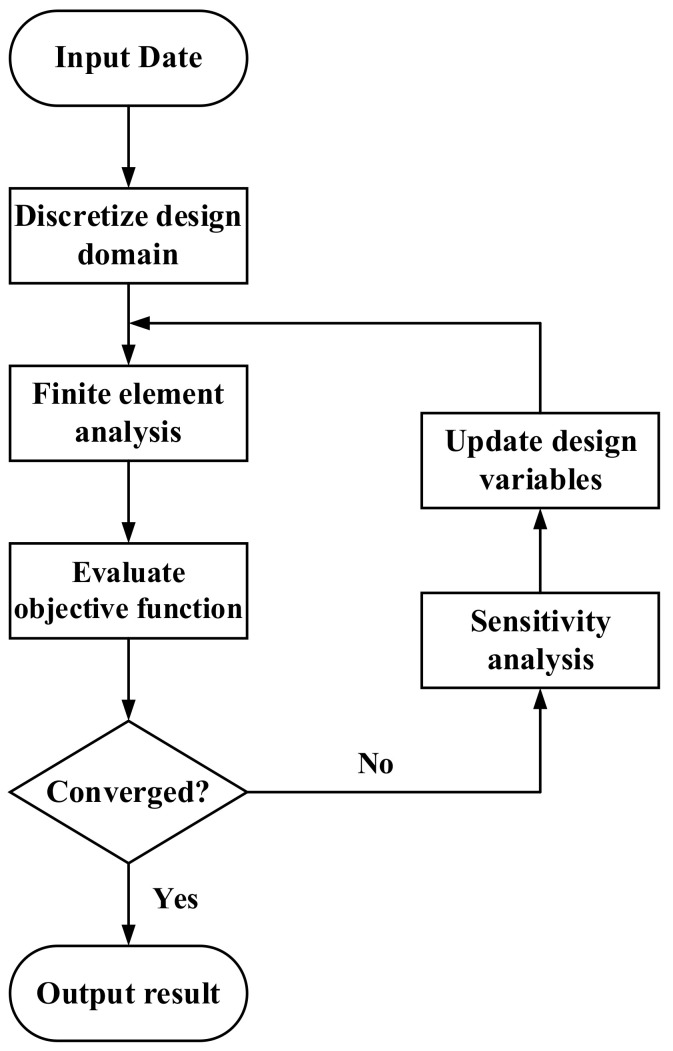
Topology optimization flowchart.

The topology optimization problem realizes the optimal design of 2D heat sink by coupling heat transfer field and flow field. In the process, the discrete design domain is completed firstly, and then the finite element analysis of flow field and temperature field is carried out by numerical method and the value of objective function is evaluated to determine whether it converges. If it does not converge, the sensitivity analysis and optimization algorithm are used to update the design variables and re-analyze them. The above process is repeated until the results converge. The topology optimization process is shown in Figure 1. The sensitivity analysis in the optimization process adopts the adjacent variable method.

### 3.1. Design Model Date and Initial Conditions 

**Figure 2:**
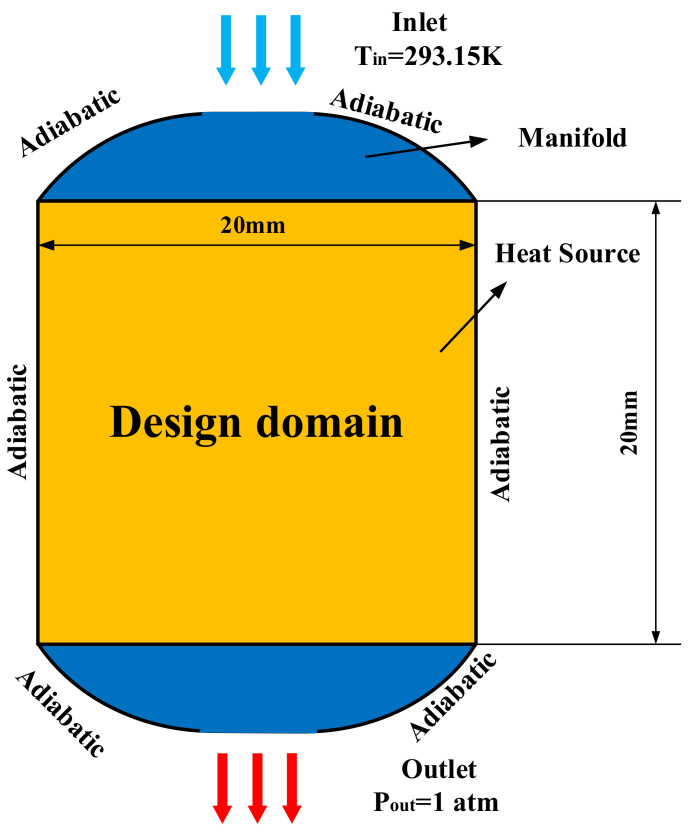
Topology optimization 2D design model.

This paper realizes the heat sink with arc-type design domain. The 2D design model is shown in Figure 2. The working fluid selected in this paper is water, while the solid material is aluminum. Table 1 is the properties of the two materials. 

In the fluid field, the flow belongs to stable laminar flow. When the fluid flows, there is no velocity slip on the channel wall. The theory that the Reynolds number affects topological structure proposed by Yaji et al. [33] is considered in the selection of velocity inlet boundary conditions. The Reynolds number can be defined as [2]:

(1) $$Re=\frac{\rho u_{fin}D_{h}}{\mu }$$

where *D_h_* is the hydraulic diameter, calculated as [2]:

(2) $$D_{h}=\frac{2W_{ch}\cdot H_{ch}}{W_{ch}+H_{ch}}$$

where *W_ch_* and *H_ch_* are the width and height of the channel, respectively.

Therefore, considering the possibility of the model, we select *R*e = 80 (*Q_v_* = 0.2 mL/s) as the initial boundary condition. The wall surface adopts an no slip boundary. At the outlet, it belongs to outlet pressure boundary condition. 

In the thermal field, as shown in Figure 2, the heat source area is a 20 × 20 mm rectangular area in which a uniform constant heat flux of 40 W/cm^2^ is applied. At the inlet, the temperature of the fluid provides a constant that equals 293.15 K. The outer boundary of the design domain adopts adiabatic boundary conditions.

### 3.2. Flow Field and Thermal Field Design

In the topological process, conjugate heat transfer is realized through the coupling of fluid and solid. The solid domain is a porous medium, while the flow belongs to the incompressible laminar flow. The flow field in topology optimization adopts the Navier–Stokes equation. Its momentum equation and continuity equation control the flow of the fluid. The equations are as follows [25]:

Continuity equation:

(3) $$\nabla \vec{u}=0$$

Flow field (Navier–Stokes equation):

(4) $$\rho \left(\vec{u}\cdot \nabla \right)\vec{u}=\nabla P+\mu \nabla^{2}\vec{u}+f$$

where *f* represents the volume force. *p*, *ρ* and *μ* represent the pressure, density and the dynamic viscosity. $\vec{u}$ represent velocity vector.

Thermal field (Energy equation):

(5) $$\rho C\left(\vec{u}\cdot \nabla T\right)=\nabla \cdot \left(\lambda \cdot \nabla T\right)+Q$$

where *λ*, *C*, and *Q* are the thermal conductivity, specific heat capacity, and heat source, respectively.

When the velocity is zero, the governing equation in the solid domain can be obtained:

(6) $$\nabla \cdot \left(\lambda \cdot \nabla T\right)+Q=0$$

### 3.3. Topology Optimization Model Design

Topology optimization is to recalculate and allocate the solid materials domain to achieve optimal heat transfer performance. In this paper, the solid isotropic microstructure with penalization (SIMP) method [34,35] and the Brinkman penalty model are used. According to SIMP, *γ* is a cell design variable, and the design domain is composed of many elements. *γ* is associated with the material parameters of the cell to control the performance of heat sink. *γ* is a number varying from 0 to 1. When *γ* = 1, it represents the fluid material. When *γ* *= 0*, it stands for the solid material. As a kind of friction force, the volume force *f* controls the flow of porous media, and *f* is defined as [27]:

(7) $$f=-\alpha \vec{u}$$

where *α* is the resistance coefficient of porous media. *α* is associated with *γ* to control the distribution of convective domain and solid domain. Therefore, the convex function in Borrvall and Peterson [21] is used to interpolate *α*:

(8) $$\alpha \left(x\right)=\alpha_{s}+\left(\alpha_{f}-\alpha_{s}\right)\gamma \left(x\right)\frac{1+p_{\alpha }}{\gamma +p_{\alpha }}$$

In this work, *α_f_ =* 0, *α_s_ =* 10^7^. When *γ* *=* 0, *α = α_s_*, it represents a solid domain. When *γ* = 1, *α = α_f_*, it represents the fluid domain. *p_α_* is the penalty factor of the resistance coefficient. The parameters of porous media *λ*, *C*, *ρ* in the heat transfer process still vary with the design variable *γ*. These parameters can be calculated by:

(9) $$\lambda \left(x\right)=\lambda_{s}+\left(\lambda_{f}-\lambda_{s}\right)\gamma \left(x\right)\frac{1+p_{\lambda }}{\gamma +p_{\lambda }}$$

(10) $$C\left(x\right)=C_{s}+\left(C_{f}-C_{s}\right)\gamma \left(x\right)\frac{1+p_{C}}{\gamma +p_{C}}$$

(11) $$\rho \left(x\right)=\rho_{s}+\left(\rho_{f}-\rho_{s}\right)\gamma \left(x\right)\frac{1+p_{\rho }}{\gamma +p_{\rho }}$$

where *f* and *s* represent the corresponding material properties of fluid and solid, respectively, *p_ρ_*, *p_λ_*, and *p_C_* represent the penalty factor of thermal conductivity, the penalty factor of density, and the penalty factor of specific heat. In the process of topology optimization, the penalty factor is used to adjust the shape of the material interpolation function, and the appropriate value can reduce the occurrence of gray cells [32].

The interpolation function of material properties affects the governing equations. The new momentum equation and temperature field equation are as follows [25]:

(12) $$\rho \left(x\right)\left(\vec{u}\cdot \nabla \right)\vec{u}=\nabla P+\mu \nabla^{2}\vec{u}-\alpha \left(x\right)\vec{u}$$

(13) $$\rho \left(x\right)C\left(x\right)\left(\vec{u}\cdot \nabla T\right)=\nabla \cdot \left(\lambda \left(x\right)\cdot \nabla T\right)+Q$$

When using SIMP for topology analysis, there may be numerical instability, such as mesh dependence, gray cells, and so on. In this paper, the Helmholtz equation is used for sensitivity filtering to reduce the mesh dependence [36].

(14) $$-r^{2}\nabla^{2}\bar{\gamma }+\bar{\gamma }=\gamma$$

where $\bar{\gamma }$ is the filter design variable, *r* is the filter radius, representing the size of a single grid cell.

To further improve numerical stability, hyperbolic tangent projection is used to avoid the generation of gray cells [37]:

(15) $$\bar{\gamma }=\frac{\left(tanh\left(\beta \left(\bar{\gamma }-\gamma_{\beta }\right)\right)+tanh\left(\beta \left(\gamma_{\beta }\right)\right)\right)}{\left(tanh\left(\beta \left(1-\gamma_{\beta }\right)\right)+tanh\left(\beta \left(\gamma_{\beta }\right)\right)\right)}$$

where *γ_β_* is the projection point, and *β* is the projection slope. 

### 3.4. Topology Optimization Objectives

As an important factor affecting the topology results, different objective functions result in different topology structures. Temperature uniformity and pressure drop minimum are the important criteria for evaluating heat sink performance, which should be considered simultaneously in the design process. Among that, the pressure drop is characterized by fluid dissipated work *Ψ*, which can be expressed by [32]:

(16) $$\Psi =\int_{\Omega }\left[\frac{1}{2}\mu \underset{i,j}{\sum }{\left(\frac{\partial u_{i}}{\partial x_{j}}+\frac{\partial u_{j}}{\partial x_{i}}\right)}^{2}+\underset{i}{\sum }\alpha \left(x\right)u_{i}{}^{2}\right]d\Omega$$

where Ω is the whole design area, *α*(*x*) is the interpolation function for the resistance coefficient of porous media.

Quantities *ϕ*_1_ and *ϕ*_2_ are two objective functions to achieve temperature uniformity, which, respectively, characterize the average temperature and temperature difference of the heat sink, which are calculated by:

(17) $$\phi_{1}=\frac{1}{\left|\Omega \right|}\int_{\Omega }Td\Omega$$

(18) $$\phi_{2}=\frac{1}{\left|\Omega \right|}\int_{\Omega }{\left(T-\phi_{1}\right)}^{2}d\Omega$$

This paper uses dual objective functions to get topology optimization design. One optimization objective is the combination of *ϕ*_1_ and *Ψ*, and the other optimization objective is the combination of *ϕ*_2_ and *Ψ*, expressed as [25]:

(19) $$П=w\frac{\phi }{\phi_{0}}+\left(1-w\right)\frac{\Psi }{\Psi_{0}}$$

where *w* is the weight occupied by their respective objectives, *ϕ*_0_ and *Ψ*_0_ are to realize the dimensionless function and represents the initial value of the objective.

The topology optimization objectives are modeled as follows to obtain the mini-channel structure [25]:

Find:

$$\gamma_{i}\left(i=1,2,\ldots ,n\right)$$

Minimize:

$$П=w\frac{\phi_{i}}{\phi_{0}}+\left(1-w\right)\frac{\Psi }{\Psi_{0}}$$

Subject to:

(20) $$\left\{\begin{array}{c} KU=F\\ \int_{\Omega }\gamma \left(x\right)d\Omega \leq f_{v}\int_{\Omega }1d\Omega \\ 0\leq \gamma_{i}\leq 1\left(i=1\ldots ...,n\right) \end{array}\right.$$

### 3.5. Topology Optimization Platform

This study uses COMSOL Multiphysics 5.4 to complete the topology optimization design process. Depending on the ref. [32], the specific topology parameters are shown in Table 2. In this paper, the Lagrange first-order linear element is used to discretize the velocity field, pressure field, and design variable field. The optimization algorithm GCMMA is used to solve the problem. This method is used to solve the large degree of freedom problem, which is very suitable for topology optimization. If the relative error of the objective function value of the two iterations in the iterative process is less than 1 × 10^−6^, it is considered that the optimization converges.

### 3.6. Topology Optimization Results

**Figure 3:**
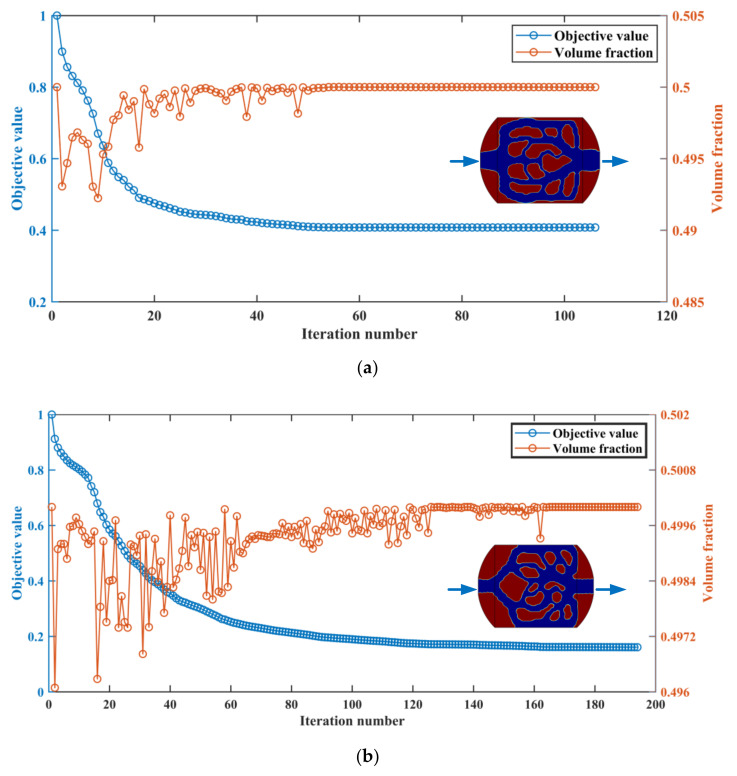
Iterative process of objective function value and volume fraction under two optimization schemes. (**a**) Minimum average temperature and fluid dissipated work; (**b**) Minimum temperature difference and fluid dissipated work.

The topology optimization results for different objective functions are shown in Figure 3. Figure 3a is the 2D topological result with minimum average temperature and fluid dissipated work as optimization objectives. The fluid domain is blue, and the solid domain is red. Figure 3b is the 2D topology result with minimum temperature difference and fluid dissipated work as optimization objectives. It can be seen that the curved design domain distributes the fluid to the channel evenly and collects the fluid to the unified outlet. 

## 4. Numerical Simulation Verification

### 4.1. Numerical Simulation Model

**Figure 4:**
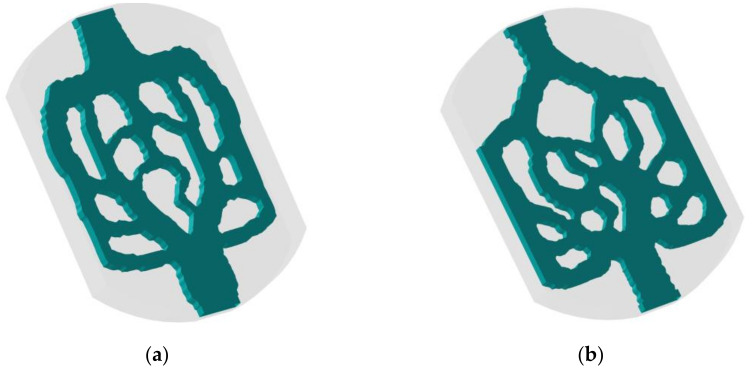
3D topology structure. (**a**) M1; (**b**) M2.

In this paper, SOLIDWORKS 2018 software is used to stretch 2D topology into the 3D structure. Figure 4 shows the internal structure of 3D topology heat sink model obtained by two different optimization objectives. The topological mini-channel (named M1) reduces the average temperature and fluid dissipation work, and the topological mini-channel (named M2) reduces the temperature difference and fluid dissipation work. The heat sink designs are composed of two arc-type design domain and a rectangular domain. The mini-channels are embedded in the design domain. 

**Figure 5:**
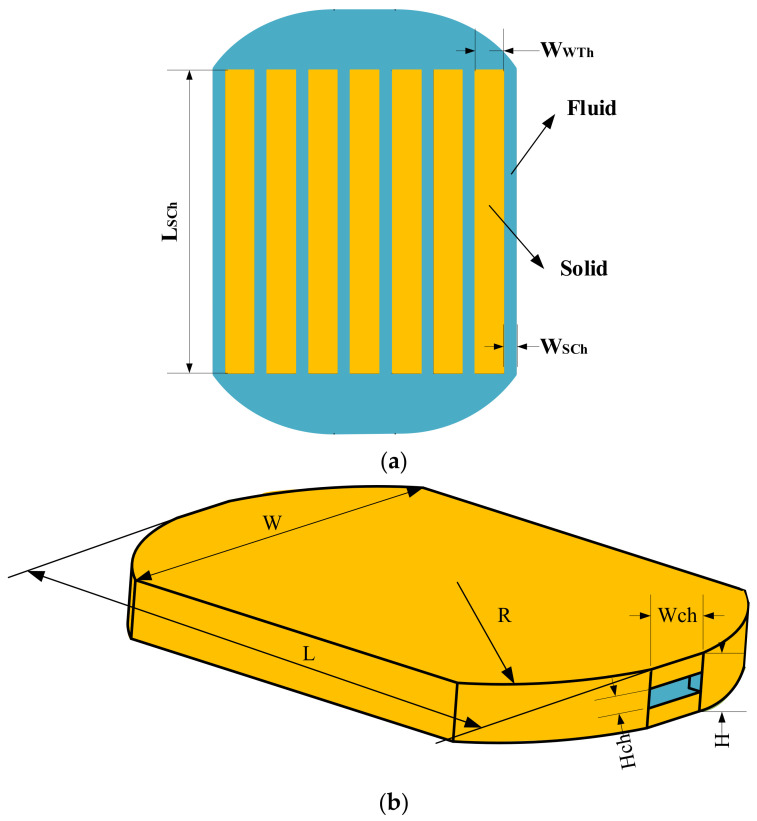
Sketch of traditional straight mini-channel heat sink. (**a**) 2D structure of straight mini-channel; (**b**) Structure of heat sink.

The comparison scheme adopted in this paper is a traditional straight mini-channel eat sink. Its boundary conditions, geometry, and fluid volume fraction are the same as topological mini-channel. Figure 5 is the three-dimensional structure of straight mini-channel heat sink (named M3), and Table 3 shows the specific dimensional parameters of heat sink. 

### 4.2. Numerical Simulation Platform

ANSYS Fluent 15.0 platform completes the numerical simulation of fluid and heat transfer characteristics of 3D heat sink (M1, M2 and M3). ICEM CFD (version 15.0) in commercial software is used to generate the mesh. The Reynolds number and volume flow rate corresponding to different flow velocities are shown in Table 4. In this paper, the solution method selected is SIMPLE, which is suitable for solving the coupling problem of pressure and velocity.

### 4.3. Numerical Simulation Governing Equation

In this paper, the following assumptions are adopted to perform the numerical simulation of three mini-channels:

1. The fluid is single phase and incompressible;
2. The flow is laminar;
3. The effects of radiation and gravity are ignored;
4. Except for the heat sink bottom plate, the others are adiabatic.

In the process of numerical simulation, the following governing equations need to be established [38]:

Continuity equation:

(21) $$\rho \nabla \vec{u}=0$$

Momentum equation:

(22) $$\rho \left(\vec{u}\cdot \nabla \right)\vec{u}=-\nabla P+\mu \nabla^{2}\vec{u}$$

Energy equation for the fluid:

(23) $$\rho C\left(\vec{u}\cdot \nabla T\right)=\nabla \cdot \left(\lambda_{f}\cdot \nabla T\right)$$

Energy equation for the solid:

(24) $$\lambda_{s}\nabla^{2}T=0$$

### 4.4. Boundary Parameter Setting

The inlet adopts a constant temperature boundary (*T_fin_* = 293.15 K), and the fluid velocity at inlet *u_fin_* is adopted:

(25) $$\vec{u}=u_{fin}=\frac{Re\cdot \mu }{\rho \cdot D_{h}}$$

The interface of fluid and solid:

(26) $$\vec{u}=0$$

The outlet adopts out pressure boundary:

(27) $$P_{out}=1\;atm$$

The Nusselt number *Nu* can be obtained by the following formula [39]:

(28) $$Nu=\frac{qD_{h}}{\lambda_{f}\left(T_{wall,bar}-T_{fbar}\right)}$$

where *T_wall,bar_* is the average wall temperature of the mini-channel, and *T_fbar_* is the average temperature of the fluid. *q* is the heat flux, and *q* = 40 W/cm^2^.

The formula for the average temperature of fluid in simulation *T_fbar_* is [27]:

(29) $$T_{fbar}=\frac{T_{fin}+T_{fout}}{2}$$

where *T_fin_* represents the inlet temperature of the fluid and *T_fout_* represents its outlet temperature.

The thermal resistance can be calculated [39]:

(30) $$R_{\mathrm{th}}=\frac{T_{surf,max}-T_{surf,min}}{q\cdot L\cdot W}$$

where *T_surf,max_* and *T_surf,min_* are the maximum heat sink bottom surface temperature and minimum heat sink bottom surface tempera

### 4.5. Grid Independence Tests

Numerical simulation needs to ensure accuracy. Three kinds of grid density, coarse, medium, and fine (4,917,623 7,046,210, and 9,092,764 elements for M1, 5,026,975 7,243,672, and 9,115,764 elements for M2, 4,028,948 6,262,361, and 8,638,427 for M3) are set, respectively. Under the same boundary conditions, the grid sensitivity tests are carried out for M1, M2, and M3, and Table 5 is the verification results. Compared with the average temperature and pressure drop under the medium density grids, the relative errors of the coarse density grids and the fine density grids of the three models are within the acceptable range, which are no more than 0.6% and 1.5%, respectively. Therefore, considering the accuracy required of simulation and the time-consuming of calculation, this paper uses the medium density grids to complete the numerical simulation.

### 4.6. Flow Characteristics Discussion 

**Figure 6:**
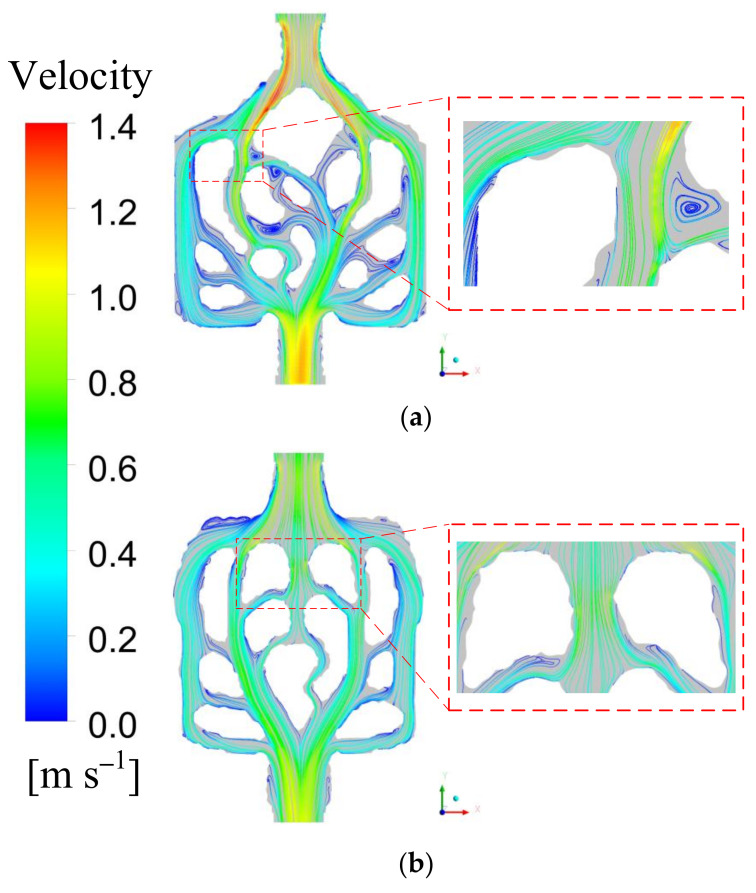
Fluid velocity distribution for M1 and M2 for *u_f_*_in_ = 0.7 m/s. (**a**) M1; (**b**) M2.

Figure 6 is the velocity vector distribution diagram of M1 and M2 at inlet speed of 0.7 m/s. It is obvious that the bifurcation and confluence, and curved corners are formed in the heat sink. When the fluid flows through the bifurcation, the velocity can be relatively evenly distributed, and the fluid forms a higher velocity distribution at the bifurcation. This means that the temperature of the fluid at the bifurcation is lower and more heat can be carried away. Therefore, the bifurcation structure affects the heat dissipation performance. The fluid in the center of the mini-channel contacts and mixes with the fluid near the wall and a secondary channel is formed between the main channel, which makes the hot working fluid and the cold working fluid mix. Therefore, the flow characteristics of the working fluid effectively improve the heat dissipation performance. It shows that the topological mini-channel structure has advantages.

**Figure 7:**
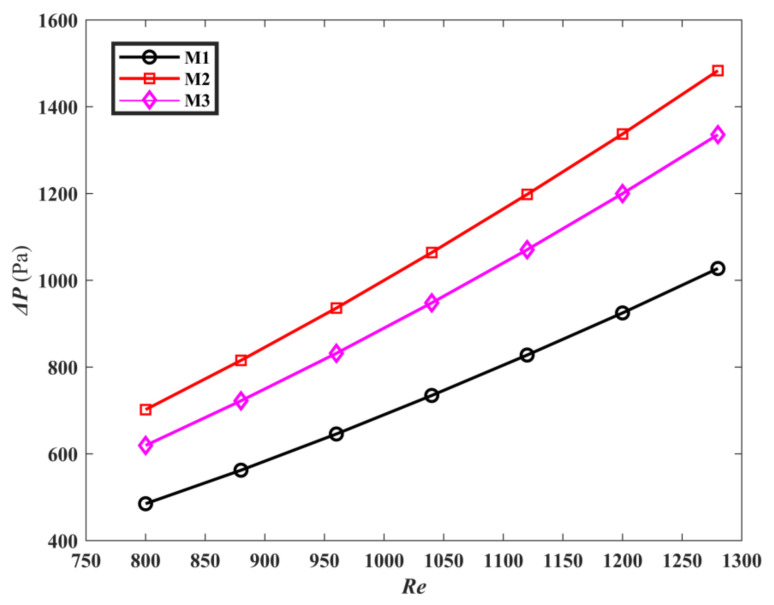
The Δ*P* of the three mini-channels varies with *Re*.

Figure 7 is comparison of the pressure drop Δ*P* of M1, M2, and M3. It is noted that as *Re* increases, the Δ*P* increases. The Δ*P* of M1 is 22.7% less than that of M3 and 30.9% less than that of M2 at the same Reynolds number. This can be explained by the fact that M1 adaptively forms circular corners between the main channels, which transfer the fluid to approach channels. This feature makes the pressure drop of M1 smaller than that of M3. Moreover, near the inlet, M1 produces more flow channels than M2, which makes the pressure drop of M1 is smaller than that of M2.

### 4.7. Thermal Characteristics Discussion 

**Figure 8:**
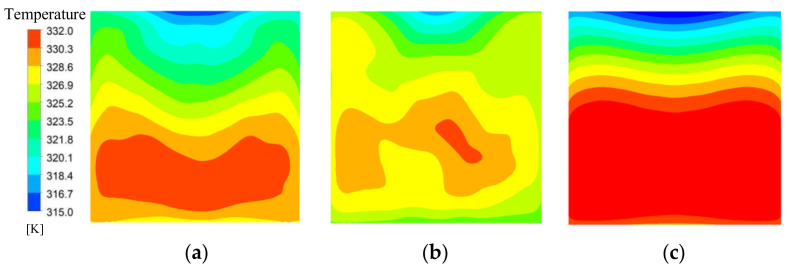
Temperature contours for *u_f_*_in_ = 0.7 m/s. (**a**) M1; (**b**) M2; (**c**) M3.

Figure 8 depicts the distribution of temperature contours on the heat sink bottom plate for M1, M2, and M3 at the flow rate of 0.7 m/s. The temperature distribution of the topological mini-channel M1 and M2 on the heat sink bottom plate is significantly lower than M3. It can be observed that a higher temperature hot zone is formed near the outlet. This is because in the process of fluid flow, the working fluid takes away heat, which increases the temperature and gradually reduces the heat exchange capacity, resulting in higher temperature at the outlet. It is noted that the temperature change of M3 is the most obvious, and M3 has more hot spots, while M1, M2 has the fewer hot spots. The temperature change for M1 and M2 is smaller. It can be explained that M1, M2 has more secondary channels than M3, which makes the fluid mixing enhanced, resulting in the reduction temperature difference of heat source surface. Therefore, the heat transfer characteristics are improved. Therefore, the topology heat sink designs have better temperature uniformity and heat dissipation performance.

**Figure 9:**
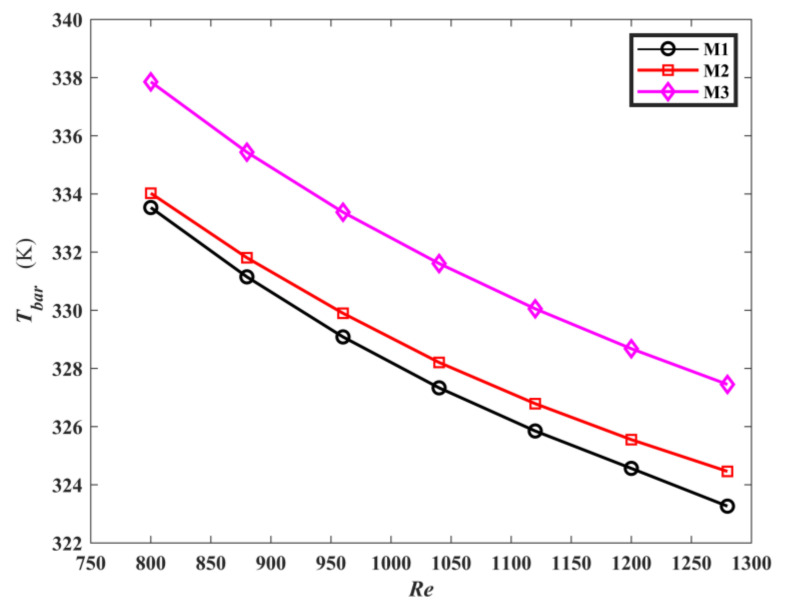
The *T_bar_* of the three mini-channels varies with *Re*.

**Figure 10:**
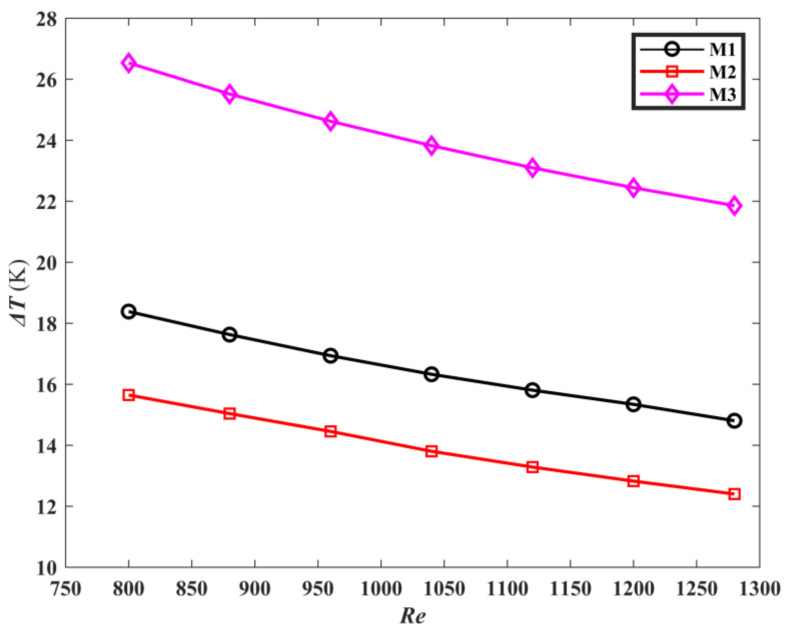
The Δ*T* of the three mini-channels varies with *Re*.

Figure 9 is the average temperature curves of the three models. As *Re* increases, the average temperature of the heat sink bottom surface *T_bar_* decreases. According to Equation (25), Reynolds number is proportional to fluid velocity. Therefore, this can be explained as the flow rate increases, and the flow heat transfer capacity enhances, resulting in the decrease of the heat sink bottom plate temperature. The average temperature of the topological mini-channel M1, M2 is lower than M3, and M1 has the lowest average temperature on the heat sink bottom plate. Figure 10 is the comparison of temperature difference on the heat sink bottom surface of M1, M2 and M3. Both of the temperature differences on the heat sink bottom surface of M1 and M2 are lower than that of M3. Compared with M3, the temperature difference of the heat sink bottom plate for M1 is reduced by 31.6%, and that of the heat sink bottom plate for M2 is reduced by 42.48%. Moreover, it is noticed that the average temperature of the heat sink bottom surface for M1 is 3.7% lower than that for M2, while the temperature difference of the heat sink bottom surface for M2 is lower than that of M1 by 15.94%. This indicates that different optimization targets produce a mini channel with different heat transfer performance. The reason is that M1 is a topological channel sink with the optimization goal of minimizing the average temperature, so that the average temperature of the heat sink bottom surface is lower compared to M2. Minimizing the temperature difference is the optimal goal for M2, Therefore, the fluid contact area of the topological channel is bigger, leading to the reduction of the temperature difference.

**Figure 11:**
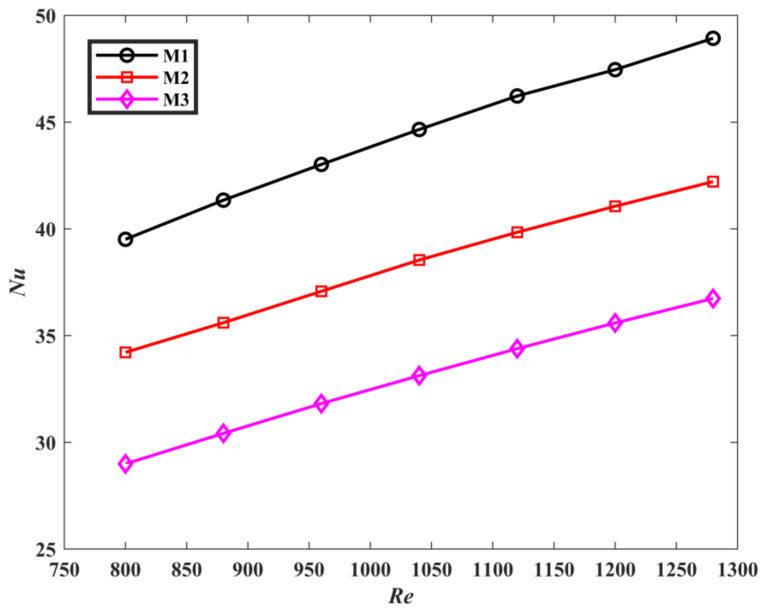
The *Nu* of the three mini-channels varies with *Re*.

**Figure 12:**
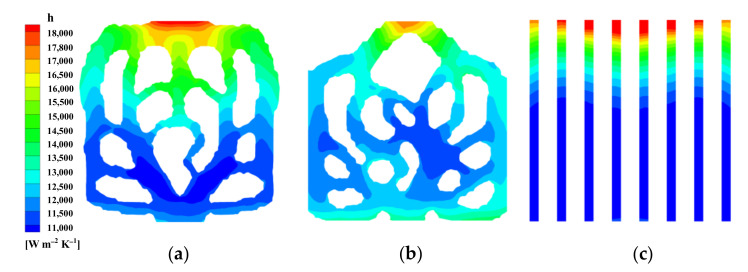
Contours of the surface heat transfer coefficient for *u_f_*_in_ = 0.7 m/s and *q* = 40 W/cm^2^. (**a**) M1; (**b**) M2; (**c**) M3.

The comparison of Nusselt number of the three mini-channels is described in Figure 11. It can be clearly noted that *Nu* of M1 and M2 is greater than M3, and M1 has the highest Nusselt number. the Nusselt numbers of M1 and M2 are increased by 34.43% and 15.86% when compared with M3, respectively. This means that the convective heat transfer ability of M1 is the strongest. This can be explained in Figure 12. Figure 12 is the local surface heat transfer coefficient *h* of the liquid-solid interface of the topological mini-channel M1, M2, and M3 at *u_fin_* = 0.7 m/s and *q* = 40 W/cm^2^. It is given by *h* = *q*/(*T*−*T_ref_*) [32,40]. *T* is the liquid-solid interface temperature, *T_ref_* = 293.15 K. In the figure, the local surface heat transfer coefficient of M3 decreases gradually. For M1 and M2, the local surface heat transfer coefficients are higher than that of M3. The surface heat transfer coefficients near the outlet are significantly higher than that of M3. The distribution of the high surface heat transfer coefficient in M1 is wider than that in M2. The surface heat transfer coefficient *h* is directly proportional to the Nusselt number *Nu*. This fully proves that M1 has better heat transfer performance.

**Figure 13:**
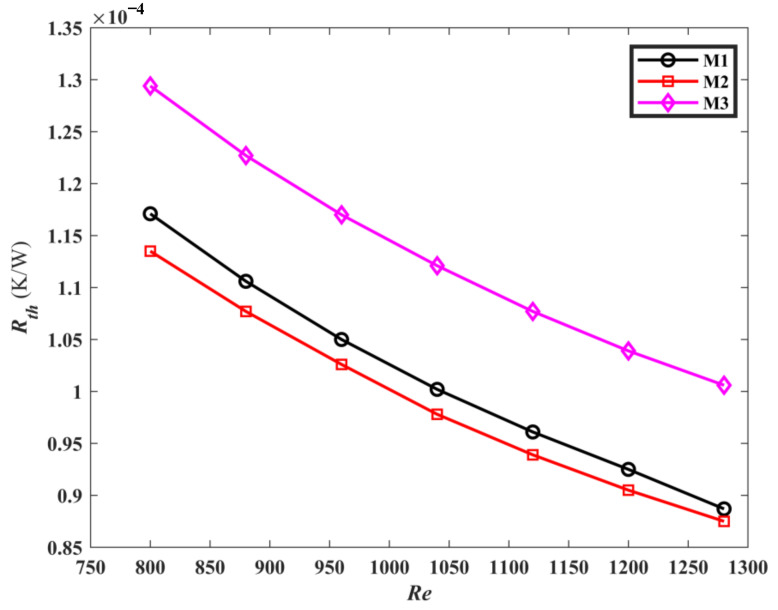
The *R_th_* of the three mini-channels varies with *Re*.

Figure 13 is the comparison of convective thermal resistance *R_th_* for M1, M2, and M3. It is noted that as *Re* increases, *R_th_* decreases. This may be interpreted as the increase of *Re*, leading to the growth of convective heat transfer, resulting in the decrease of *R_th_* between solid wall and fluid. Furthermore, because the thermal resistance in the process of heat conduction is constant, the total thermal resistance is reduced. Compared with M3, the thermal resistances of M1 and M2 are decreased by 10.8% and 12.8% at the same Reynolds number, respectively. This reflects that the topological mini-channel has a stronger heat transfer ability.

### 4.8. Performance Valuation Discussion

In this paper, the overall performance of M1, M2 and M3 is comprehensively evaluated by pressure drop Δ*P* and Nusselt number *Nu*. *PEC* can be defined as the thermal performance evaluation criterion. *PEC* is calculated as [41]:

(31) $$PEC=\frac{Nu_{T}/Nu_{s}}{\sqrt[{3}]{\Delta T_{T}/\Delta T_{S}}}=\frac{E_{Nu}}{\sqrt[{3}]{E_{\Delta P}}}$$

where *Nu_T_* represents *Nu* of the topological heat sink M1 and M2, *Nu_S_* represents *Nu* of M3. Δ*P_T_* and Δ*P_S_* are Δ*P* of the topological heat sink and the conventional straight mini-channel, respectively. *E_Nu_* is the average heat transfer enhancement, and *E*_Δ*P*_ denotes the pressure drop improvement.

**Figure 14:**
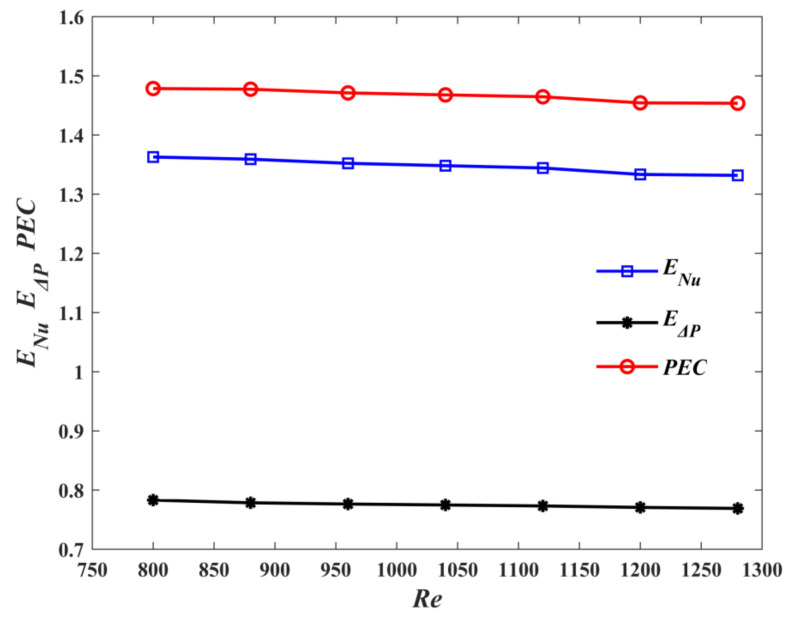
Effect of *Re* on *E_Nu_*, *E*_Δ*P*_ and *PEC* for M1.

**Figure 15:**
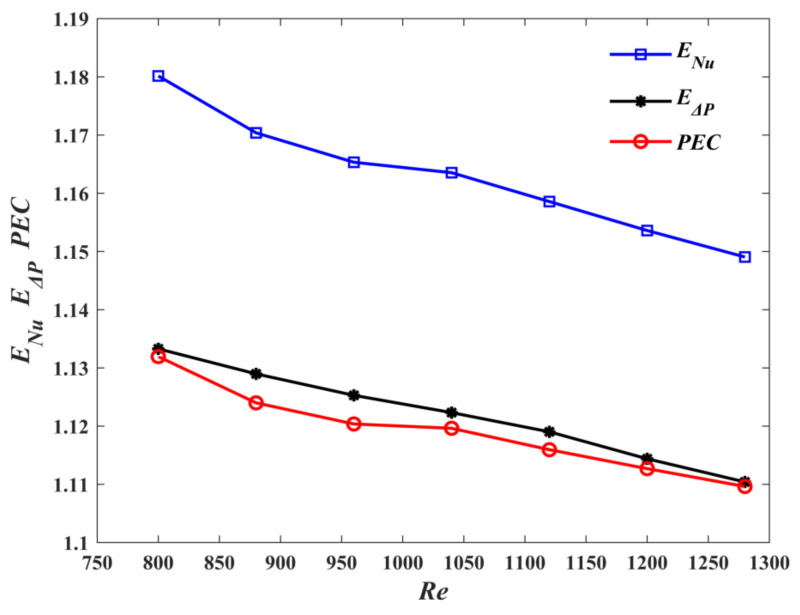
Effect of *Re* on *E_Nu_*, *E*_Δ*P*_ and *PEC* for M2.

Figure 14 shows the variations of *E_Nu_*, *E*_Δ*P*_, and *PEC* with *Re* for M1 and M3. With the increase of *Re*, *E*_Δ*P*_ > 1. *PEC* decreases slightly, while *PEC* > 1. It fully indicates that the M1 is superior to the M3 in terms of overall thermal performance. Figure 15 demonstrates the variations of *E_Nu_*, *E*_Δ*P*_ and *PEC* with Reynolds number. As shown in the figure, *E*_Δ*P*_ > 1. This can be explained by the fact that, compared, with M3, M2 has a larger flow length and friction with the wall, which leads to the increase of pressure drop. However, *E_Nu_* > 1 indicates that the heat transfer performance of M2 is better than that of M3. The *PEC* values are >1, which illustrates that the negative effect of the increase of pressure drop is overcome by the more dominant positive effect of the increase of heat transfer, which makes the overall performance of M2 better than that of M3. 

## 5. Experimental Verification

### 5.1. Model Manufacturing

**Figure 16:**
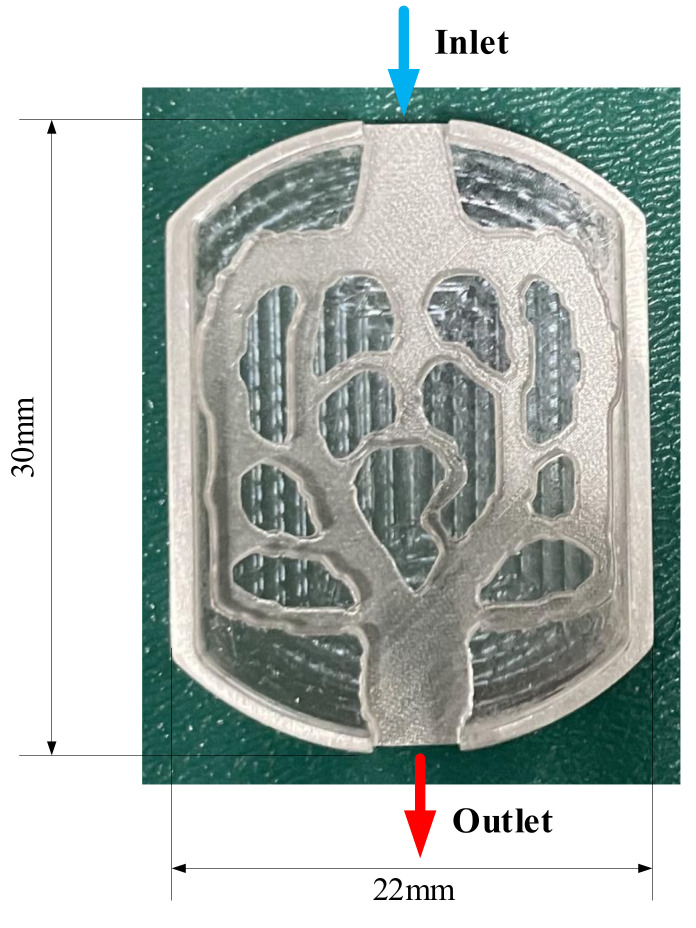
Experimental heat sink.

Through the comprehensive analysis of M1 and M2, it was found that M1 has more advantages in heat dissipation. M1 was selected for the experimental test to verify the accuracy of simulation results. Given the small size of the model, 3D printing was selected to process the model. The processing material was aluminum, and the sample is shown in Figure 16.

### 5.2. Experimental Procedure and Apparatus

**Figure 17:**
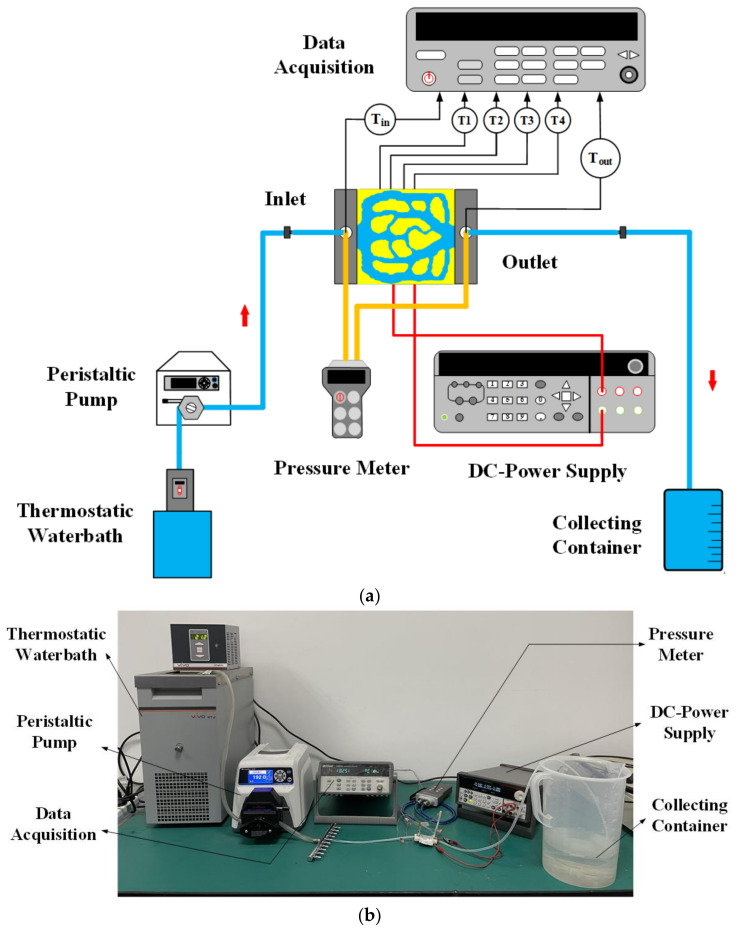
Verification device of numerical simulation results. (**a**) Experimental schematic diagram; (**b**) Experimental test system.

The experimental schematic diagram is shown in Figure 17a. Figure 17b describes the testing apparatus. The fluid was supplied to the topology min-channel heat sink by the peristaltic pump for heat dissipation through the thermostatic water bath. The water was recovered at the collecting container. The function of the thermostatic water bath (JULABO-VIVO RT2) was maintaining the inlet temperature at 293 K. The boundary condition of the velocity inlet was realized by the peristaltic pump (Masterflex GY7792175), and the inlet flow could be controlled by adjusting pumping parameters. In this experiment, the peristaltic pump was used to provide the volume flow of 3.0–3.2 mL/s for M1. To simulate the boundary condition of the heat source, two thin-film resistors were installed on the bottom surface of the heat sink, and a layer of silicon dioxide with a conduction coefficient of 2.1 W/m∙K was added between the bottom surface and the thin film resistor to eliminate the contact thermal resistance. The rated power of each thin film resistor (MP9100-274) was 100 W, the resistance is 20 Ω, and the current was 0–5 A. The DC power supply (2230G-30-6) provides the heat flow of 40 W/cm^2^. The wall temperature was measured by four K-type thermocouples with diameter of 0.5 mm. The temperature of the inlet and outlet is obtained by placing two K-type thermocouples in slots at the inlet and outlet. The data acquisition (Model 34972A, Agilent Technologies, Guangdong, China) displays temperature readings. Similarly, the pressure drop is measured by inserting the pipeline of the digital pressure gauge (Comark C9555) into the inlet and outlet slot. In order to improve the accuracy of experimental verification, in this experiment, the thermal insulation boundary conditions in numerical simulation were realized by wrapping the heat sink in thermal insulation materials.

### 5.3. Experimental Post-Treatment

#### 5.3.1. Wall Temperature Calculation

**Figure 18:**
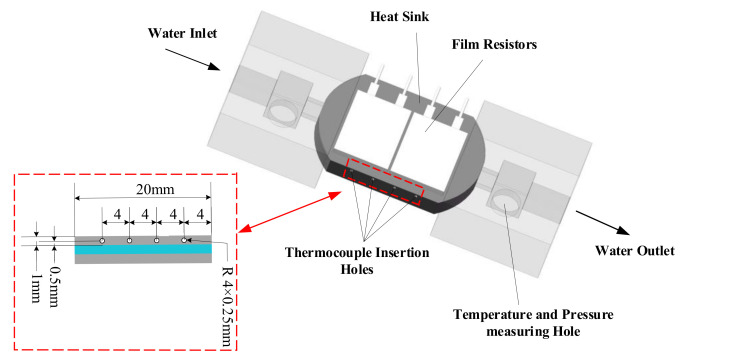
Assembly drawing of the heat sink (**right**) and location of four temperature measuring holes (**left**).

In the experiment, the wall temperature cannot be measured directly by instruments and equipment. In this experiment, the channel wall temperature is indirectly obtained by machining temperature measuring holes on the outer surface. The four measuring points are 0.5 mm away from the channel wall. Figure 18 depicts the assembly drawing of M1 and the position distribution of the four thermocouples. In order to improve the accuracy of temperature measurement, thermal conductive adhesive is applied to the holes in this experiment. In this paper, the local wall temperature could be achieved by one-dimensional heat conduction law. Therefore, the wall temperature *T_wtci_* can be calculated [42]:

(32) $$T_{wtic}=T_{ytic}-\frac{P_{in}\cdot y}{A_{eff}\lambda_{s}}$$

where *T_ytci_* is the temperature readings of the thermocouples by the experiment, *y* = 0.5 mm. *A_eff_* is the heat transfer area. 

After obtaining the local temperatures of the four locations, this paper used the average of the four temperature values to calculate the average temperature of the wall *T_wall,avg_*:

(33) $$T_{wall,avg}=\frac{\sum_{i=1}^{4}T_{wtic}}{4}$$

#### 5.3.2. Uncertainty Calculation

In the experiment, there was uncertainty when manufacturing the model and measuring the temperature and pressure drop through the instrument. Table 6 shows the uncertainty of measurement parameters obtained from model machining accuracy and instrument manual. In this paper, the formula of performance parameters uncertainty is obtained by the Coleman method [43] and ASME standard [44], calculated as:

(34) $$U_{R}={\left[\sum_{i=1}^{n}{\left(\frac{\partial R}{\partial V_{i}}U_{V_{i}}\right)}^{2}\right]}^{1/2}$$

where *U_Vi_* is the absolute uncertainty of the parameters, and *n* is the number of parameters.

Combined with Appendix A and Table 6, we calculate that the uncertainty (*U_Nu_*/*Nu*) × 100% of *Nu* is 3.7%.

#### 5.3.3. Experimental Result Analysis

In order to verify the accuracy of M1 numerical results, the same boundary conditions as the numerical simulation were achieved in this experiment. Table 7 lists the pressure drop and the average temperature data and errors of M1 obtained by numerical simulation and experiment at different speeds. It can be observed that relative error of Δ*P* did not exceed 5.0%, and the relative error of *T_wall,bar_* was within 1.7%. It fully indicates that the experimental data and simulation data are consistent.

**Figure 19:**
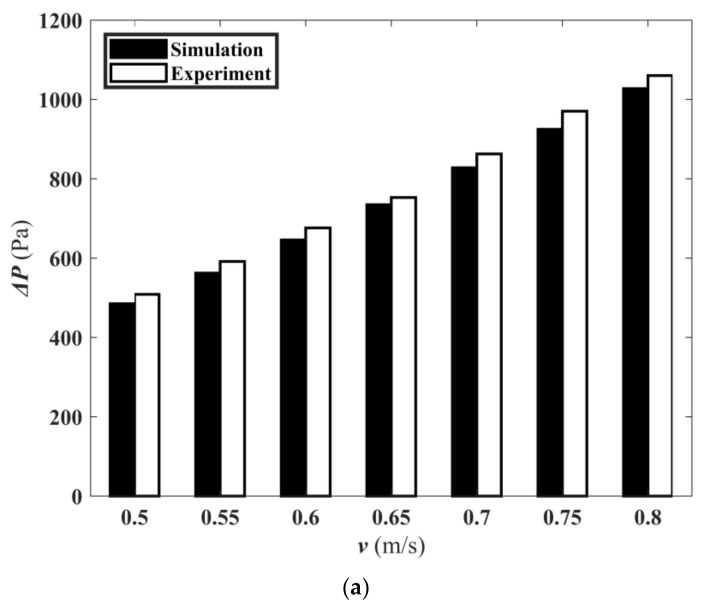

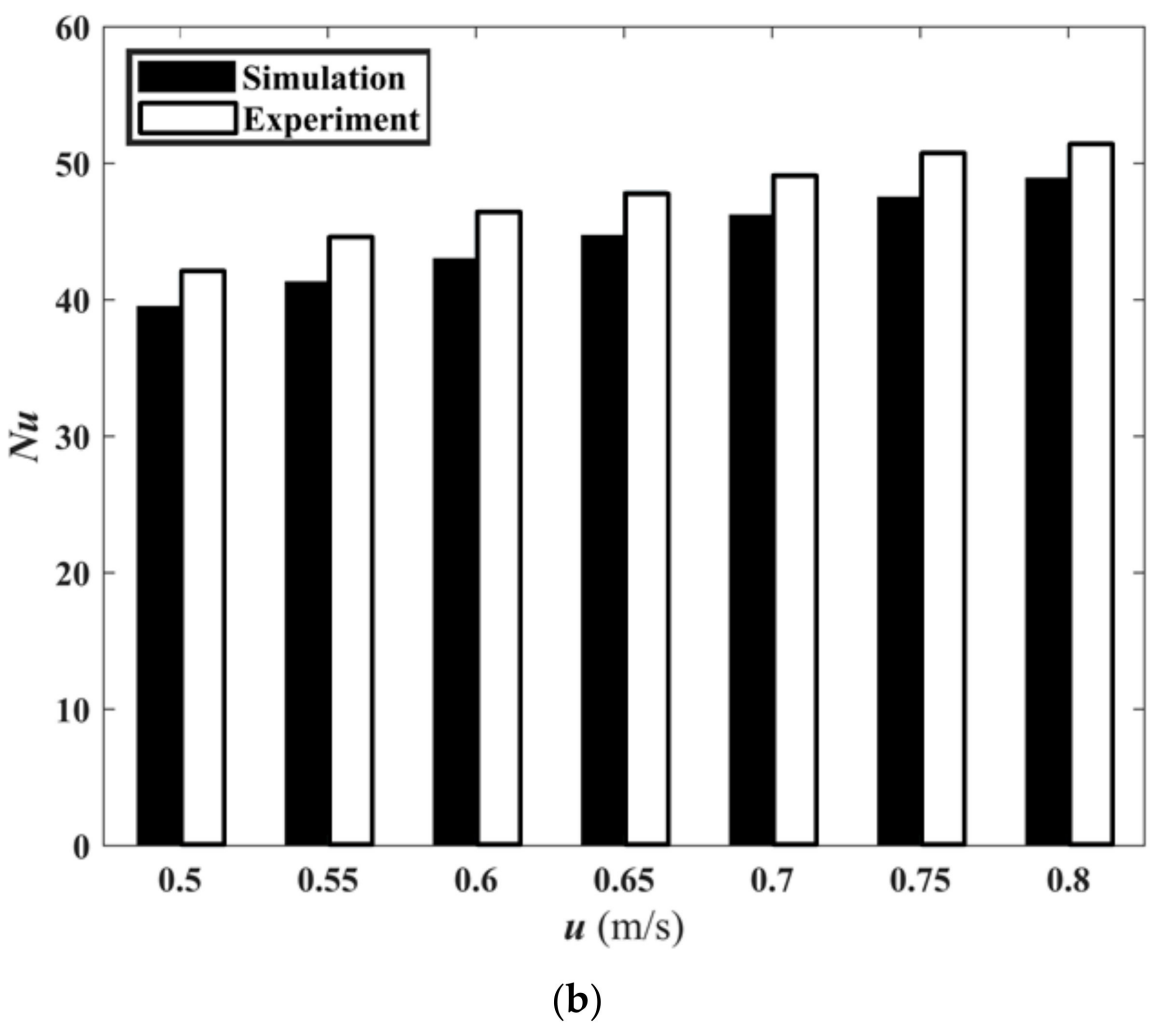
Experimental and simulation results. (**a**) The Δ*P_sim_* and Δ*P_exp_* of M1 with flow rate; (**b**) Simulated Nusselt number and experimental Nusselt number of M1 with flow rate.

Figure 19a shows the simulated and measured pressure drop at different speeds. Figure 19b is the histogram of the Nusselt number with velocity obtained from simulation and experiment. The relative error between the simulation and experiment results was less than 7.5%. This indicated that the experimental results are reliable.

## 6. Conclusions

In this paper, two kinds of mini-channel heat sinks with high heat transfer capacity are obtained by using topology optimization technology. The flow and heat transfer characteristics are studied and compared with traditional straight mini-channel heat sinks. Some conclusions can be drawn:

1. Compared with the traditional straight mini-channel heat sink, the topological mini-channel heat sink design had better flow and heat transfer performances.
2. Compared with M2, the Δ*P* of M1 was decreased by 30.9%. Compared with M3, the Δ*T* of M1 and M2 were decreased by 31.6% and 42.48%, respectively.
3. Compared with M3, the *Nu* of M1 and M2 were increased by 34.43% and 15.86%, respectively. Compared with M3, the *R_th_* of M1 and M2 were decreased by 10.8% and 12.8%, respectively.
4. M1 is the best design for flow and heat transfer performance.
5. The simulation results agree well with the experimental results.

## Figures and Tables

**Table 1:** Material properties.

Material	*ρ* (kg/m^3^)	*C* (J/kg·K)	*λ* (W/m·K)	*μ* (Pa·s)
Aluminum	2719	871	202.4	-
Water	998.2	4182	0.62	1 × 10^−3^

**Table 2:** Model topological parameters.

Parameter	Value	Parameter	Value
*p_α_*	0.02	*γ_β_*	0.5
*p_ρ_*	0.01	*β*	10
*p_λ_*	0.01		
*p_C_*	100		

**Table 3:** Model specific dimensional parameters.

Parameter	Value	Parameter	Value
*H* (mm)	3	*R* (mm)	14.14
*H_ch_* (mm)	1	*W_WTh_* (mm)	1.83
*L* (mm)	30	*W_SCh_* (mm)	0.9
*W_ch_* (mm)	4	*L_SCh_* (mm)	20
*W* (mm)	22		

**Table 4:** Reynolds number at different flow velocities.

*v* (m/s)	*Q_v_* (mL/s)	*Re*
0.5	2.0	800
0.55	2.2	880
0.6	2.4	960
0.65	2.6	1040
0.7	2.8	1120
0.75	3.0	1200
0.8	3.2	1280

**Table 5:** Grid independence test result.

Model	Grids	*T_bar_* (K)	Error (%)	Δ*P* (Pa)	Error (%)
M1	4,917,623	335.276	0.52	491.437	1.3
	7,046,210	333.533	-	485.049	-
	9,092,764	332.270	0.38	480.865	0.87
M2	5,026,975	335.838	0.54	723.521	1.27
	7,243,672	334.025	-	714.333	-
	9,115,764	332.793	0.37	708.312	0.85
M3	4,028,948	339.963	0.62	628.438	1.5
	6,262,361	337.856	-	619.012	-
	8,638,427	336.004	0.55	611.671	1.2

**Table 6:** Uncertainty data of parameters.

Parameter	Absolute Uncertainty	Relative Uncertainty
Channel width (*W)*	±0.01 mm	-
Channel height (*H*)	±0.01 mm	-
Temperature (*T*)	±0.2 K	-
Power (*P_in_*)	-	±0.2%
Pressure drop (Δ*P*)	-	±0.3%
Volume flow rate (*Q_v_*) Nusselt number (*Nu*)	-	±0.1%
	-	3.7%

**Table 7:** Comparison of experimental and numerical simulation data at different flow velocities.

*v* (m/s)	Δ*P_(sim)_* (Pa)	Δ*P_(exp)_* (Pa)	Error (%)	*T_wall,bar(sim)_* (K)	*T_wall,avg(exp)_* (K)	Error (%)
0.5	485.0	508.7	4.6	333.5	329.6	1.2
0.55	562.3	591.6	4.9	331.2	327.8	1.0
0.6	645.8	676.2	4.5	329.1	325.6	1.1
0.65	734.6	752.8	2.4	327.3	323.5	1.2
0.7	827.8	862.7	4.0	325.9	321.2	1.5
0.75	924.8	970.4	4.7	324.6	320.3	1.4
0.8	1027.3	1060.2	3.1	323.3	317.8	1.7

## Nomenclature

*A_eff_*	Effective heat transfer area, m^2^
*C*	Diameter of the pipe, m
*D_h_*	Hydraulic diameter, m
*E_Nu_*	Average Nusselt number enhancement
*E_ΔP_*	Pressure drop enhancement
*H*	Height of heat sink, m
*H_ch_*	Height of channel, m
*h*	The local surface heat transfer coefficient, W/m^2^∙K
*L*	Length of heat sink, m
*L_SCh_*	Length of straight channel, m
*L_WTh_*	Wall thickness of straight channel, m
*Nu*	Nusselt number
*p_α_*	Penalty factor of resistance coefficient
*p_λ_*	Penalty factor of thermal conductivity
*p_C_*	penalty factor of specific heat capacity
*p_ρ_*	Penalty factor of density
*P_p_*	Pump power, W
*Q_v_*	Volume flow rate, mL/s
*Q_in_*	Total heat input, W
*R*	Design domain radius, m
*Re*	Reynolds number
*Rth*	Thermal resistance, K/W
*r*	Filter radius, m
*T_bar_*	Average substrate temperature, K
*T_wall,bar_*	Average wall temperature, K
*T_fbar_*	Average fluid temperature, K
*T_surf,max_*	Maximum substrate temperature, K
*T_ytci_*	Thermocouple temperature, K
*T_wtci_*	Wall temperature by calculating, K
*u_fin_*	Fluid velocity at inlet, m/s
*W*	Width of heat sink, m
*W_ch_*	Width of channel, m
*W_SCh_*	Width of straight channel, m
*α*	Resistance coefficient of porous media
*β*	Projection slope
*γ*	Design variable
$\bar{\gamma }$	Filter design variable
*λ*	Thermal conductivity, W/m∙K
*ρ*	Density, kg/m^3^
*ϕ_1_*	Average temperature function, K
*ϕ_2_*	Temperature difference function, K^2^
*μ*	Dynamic viscosity of fluid, Pa∙s
*Π*	Double objective function
*f*	Fluid
*s*	Solid
*avg*	Average
*max*	Maximum
*min*	Minimum
*tci*	Thermocouple location

## Appendix A

According to Equations (28) and (34), the relative uncertainty *U_Nu_*/*Nu* is calculated as follows:

(A1) $$\frac{U_{Nu}}{Nu}=\sqrt{{\left(\frac{U_{q}}{q}\right)}^{2}+{\left(\frac{U_{D_{h}}}{D_{h}}\right)}^{2}+{\left(\frac{U_{\Delta T}}{\Delta T}\right)}^{2}+{\left(\frac{U_{\lambda_{f}}}{\lambda_{f}}\right)}^{2}}$$

(A2) $$U_{\Delta T}=\sqrt{{\left(T_{wavg}\right)}^{2}+{\left(T_{favg}\right)}^{2}}$$

(A3) $$U_{T_{wavg}}=\frac{\sqrt{{\left(U_{T_{wtc1}}\right)}^{2}+{\left(U_{T_{wtc2}}\right)}^{2}+{\left(U_{T_{wtc3}}\right)}^{2}+{\left(U_{T_{wtc4}}\right)}^{2}}}{4}$$

(A4) $$U_{T_{favg}}=\frac{\sqrt{{\left(T_{fin}\right)}^{2}+{\left(T_{fout}\right)}^{2}}}{2}$$

(A5) $$U_{D_{h}}=\sqrt{{\left(\frac{2H}{W+H}U_{W}+\frac{-1}{\left(W^{2}+H^{2}\right)}2HWU_{W}\right)}^{2}+{\left(\frac{2W}{W+H}U_{H}+\frac{-1}{\left(W^{2}+H^{2}\right)}2HWU_{H}\right)}^{2}}$$

## References

[1] Yang L., Huang J.N., Mao M., Jia W.K. (2020). Numerical assessment of Ag-water nano-fluid flow in two new microchannel heatsinks: Thermal performance and thermodynamic considerations. Int. Commun. Heat Mass Transf..

[2] Liu H.L., Qi D.H., Shao X.D., Wang W.D. (2019). An experimental and numerical investigation of heat transfer enhancement in annular microchannel heat sinks. Int. J. Therm. Sci..

[3] Wang G.L., Qian N., Ding G. (2019). Heat transfer enhancement in microchannel heat sink with bidirectional rib. Int. J. Heat Mass Transf..

[4] Ghani I.A., Sidik N.A.C., Kamaruzaman N. (2017). Hydrothermal performance of microchannel heat sink: The effect of channel design. Int. J. Heat Mass Transf..

[5] Zhang C.P., Lian Y.F., Yu X.F., Liu W.J., Teng J.T., Xu T.T. (2013). Numerical and experimental studies on laminar hydrodynamic and thermal characteristics in fractal-like microchannel networks. Part A: Comparisons of two numerical analysis methods on friction factor and Nusselt number. Int. J. Heat Mass Transf..

[6] Chen H.H. (2007). Forced convection heat transfer in microchannel heat sinks. Int. J. Heat Mass Transf..

[7] Zhao C.Y., Lu T.J. (2002). Analysis of microchannel heat sinks for electronics cooling. Int. J. Heat Mass Transf..

[8] Li J., Peterson G.P., Cheng P. (2004). Three-dimensional analysis of heat transfer in a micro-heat sink with single phase flow. Int. J. Heat Mass Transf..

[9] Al-Neama A.F., Kapur N., Summers J., Thompson H.M. (2017). An experimental and numerical investigation of the use of liquid flow in serpentine microchannels for microelectronics cooling. Appl. Therm. Eng..

[10] Bi B., Tang G.H., Tao W.Q. (2013). Heat transfer enhancement in microchannel heat sinks with dimples and cylindrical grooves. Appl. Therm. Eng..

[11] Zhou F., Zhou W., Zhang C.Y., Qiu Q.F., Yuan D., Chu X.Y. (2020). Experimental and numerical studies on heat transfer enhancement of microchannel heat sink embedded with different shape micropillars. Appl. Therm. Eng..

[12] Zhang Y., Pan M. (2007). Simulation Analysis of the Heat Transfer Performance of an N-type Microchannel Heat sink. Chem. Eng. Technol..

[13] Moradikazerouni A., Shoele K. (2021). Computational study of Rayleigh-Bernard convection in a cylindrical pressurized cryogenic tank. Bull. Am. Phys. Soc..

[14] Ma Y.L., Shahsavar A., Moradi I., Rostami S., Moradikazerouni A., Yarmand H. (2021). Using finite volume method for simulating the natural convective heat transfer of nano-fluid flow inside an inclined enclosure with conductive walls in the presence of a constant temperature heat source. Physica A.

[15] Estebe C., Liu Y., Vahab M. A Low Mach Number, Adaptive Mesh Method for Simulating Multi-phase Flows in Cryogenic Fuel Tanks. Proceedings of the SIAM Conference on Computational Science and Engineering.

[16] Moradikazerouni A., Vahab M., Shoele K. A 0D/3D nodal-CFD method of cylindrical pressurized tanks. Proceedings of the 73rd Annual Meeting of the APS Division of Fluid Dynamics.

[17] Lu S., Vafai K. (2016). A comparative analysis of innovative microchannel heat sinks for electronic cooling. Int. Commun. Heat Mass Transf..

[18] Deng D., Wan W., Tang Y., Shao H., Huang Y. (2015). Experimental and numerical study of thermal enhancement in reentrant copper microchannels. Int. J. Heat Mass Transf..

[19] Moradikazerouni A., Afrand M., Alsarraf J. (2019). Investigation of a computer CPU heat sink under laminar forced convection using a structural stability method. Int. J. Heat Mass Transf..

[20] Bendsøe M.P., Kikuchi N. (1988). Generating optimal topologies in structural design using a homogenization method. Comput. Meth. Appl. Mechanics Eng..

[21] Borrvall T., Petersson J. (2003). Topology optimization of fluids in stokes flow. Int. J. Numer. Methods Fluids..

[22] Dede E.M. Multiphysics topology optimization of heat transfer and fluid flow systems. Proceedings of the COMSOL Conference.

[23] Zhang B., Zhu J.H. (2020). Topology optimization design of nanofluid-cooled microchannel heat sink with temperature-dependent fluid properties. Appl. Therm. Eng..

[24] Joo Y., Lee I., Kim S.J. (2018). Efficient three-dimensional topology optimization of heat sinks in natural convection using the shape-dependent convection model. Int. J. Heat Mass Transf..

[25] Han X.H., Liu H.L., Xie G.N., Sang L., Zhou J.Z. (2021). Topology optimization for spider web heat sinks for electronic cooling. Appl. Therm. Eng..

[26] Qiu G.Q., Wei P., Huang P.N., Pan M.Q. (2021). Topology Optimization Design of a Microchannel Plate Based on Velocity Distribution. Chem. Eng. Technol..

[27] Zhou T., Chen B.C., Liu H.L. (2021). Study of the Performance of a Novel Radiator with Three Inlets and One Outlet Based on Topology Optimization. Micromachines.

[28] Liu H.L., An X.K., Wang S.C. (2017). Heat transfer performance of T-Y type micro-channel heat sink with liquid GaInSn coolant. Int. J. Therm. Sci..

[29] Wang J.Y. (2015). Theory and practice of flow field designs for fuel cell scaling-up: A critical review. Appl. Therm. Eng..

[30] Vinodhan V.L., Rajan K. (2015). Computational analysis of new microchannel heat sink configurations. Energy Convers. Manag..

[31] Li H., Ding X., Jing D., Xiong M., Meng F. (2019). Experimental and numerical investigation of liquid-cooled heat sinks designed by topology optimization. Int. J. Therm. Sci..

[32] Hu D.H., Zhang Z.W. (2020). Numerical study on flow and heat transfer characteristics of microchannel designed using topological optimizations method. Technol. Sci..

[33] Yaji K., Yamada T. (2015). A topology optimization method for a coupled thermal–fluid problem using level set boundary expressions. Int. J. Heat Mass Transf..

[34] Bendsøe M.P. (1989). Optimal shape design as a material distribution problem. Struct. Optim..

[35] Bruns T.E. (2007). Topology optimization of convection-dominated, steady-state heat transfer problems. Int. J. Heat Mass Transf..

[36] Lazarov B.S., Sigmund O. (2010). Filters in topology optimization based on Helmholtz-type differential equations. Int. J. Numer. Methods Eng..

[37] Wang F., Lazarov B.S., Sigmund O. (2011). On projection methods, convergence and robust formulations in topology optimization. Struct. Multidiscip. Optim..

[38] Patankar S.V. (1980). Numerical Heat Transfer and Fluid Flow.

[39] Liu H.L., Yao Y., Xie G.N., Xie Z.L. (2021). Improved thermal performance of new staggered double P-type minichannel heat exchangers. Appl. Therm. Eng..

[40] Wang X.Q., Mujumdar A.S., Yap C. (2006). Thermal characteristics of tree-shaped microchannel nets for cooling of a rectangular heat sink. Int. J. Therm. Sci..

[41] Gong L., Kota K., Tao W.Q., Joshi Y. (2011). Thermal performance of microchannels with wavy walls for electronics cooling. IEEE Trans. Compon. Packag. Manufac. Technol..

[42] Lee Y.J., Lee P.S., Chou S.K. (2012). Enhanced thermal transport in microchannel using oblique fins. J. Heat Transfer..

[43] Schultz R., Cole R. (1979). Uncertainty analysis in boiling nucleation. Am. Inst. Chem. Eng..

[44] ASME (2005). PTC 19.1-2013. (Revision of ASME PTC 19.1-2005). Test Uncertainty.

